# 3,6-Dibromo-9-(4-*tert*-butyl­benz­yl)-9*H*-carbazole

**DOI:** 10.1107/S1600536808022174

**Published:** 2008-07-23

**Authors:** Duan-Lin Cao, Jian-Lan Cui, Wei Mao

**Affiliations:** a School of Chemical Engineering and Technology, Tianjin University, Tianjin 300072, People’s Republic of China; b Department of Chemistry and Environment, North University of China, Taiyuan 030051, People’s Republic of China

## Abstract

In the title compound, C_23_H_21_Br_2_N, which was synthesized by the *N*-alkyl­ation of 1-*tert*-butyl-4-(chloro­meth­yl)benzene with 3,6-dibromo-9*H*-carbazole, the asymmetric unit contains two unique mol­ecules. Each carbazole ring system is essentially planar, with mean deviations of 0.0077 and 0.0089 Å for the two mol­ecules. The carbazole planes make dihedral angles of 78.9 (2) and 81.8 (2)° with the planes of the respective benzene rings.

## Related literature

For the pharmaceutical properties of carbazole derivatives, see: Buu-Hoï & Royer (1950 ▶); Caulfield *et al.* (2002 ▶); Harfenist & Joyner (1983 ▶); Harper *et al.* (2002 ▶). For the preparation of the title compound, see: Duan *et al.* (2005 ▶); Smith *et al.* (1992 ▶). For reference structural data, see: Allen *et al.* (1987 ▶).
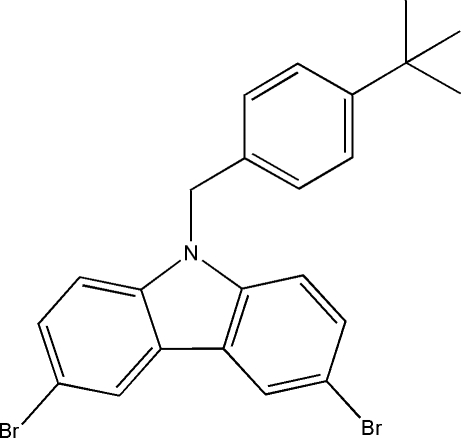


## Experimental

### 

#### Crystal data

C_23_H_21_Br_2_N
*M* *_r_* = 471.23
Triclinic, 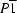

*a* = 11.240 (2) Å
*b* = 12.921 (3) Å
*c* = 15.694 (3) Å
α = 105.43 (3)°
β = 108.53 (3)°
γ = 103.09 (3)°
*V* = 1958.9 (10) Å^3^
*Z* = 4
Mo *K*α radiation
μ = 4.14 mm^−1^
*T* = 113 (2) K
0.10 × 0.08 × 0.04 mm

#### Data collection

Rigaku Saturn CCD diffractometer
Absorption correction: multi-scan (*CrystalClear*; Rigaku/MSC, 2005 ▶) *T*_min_ = 0.682, *T*_max_ = 0.852
12085 measured reflections
6891 independent reflections
4712 reflections with *I* > 2σ(*I*)
*R*_int_ = 0.051

#### Refinement

*R*[*F* ^2^ > 2σ(*F* ^2^)] = 0.055
*wR*(*F* ^2^) = 0.112
*S* = 1.01
6891 reflections
475 parameters
H-atom parameters constrained
Δρ_max_ = 0.46 e Å^−3^
Δρ_min_ = −0.46 e Å^−3^

### 

Data collection: *CrystalClear* (Rigaku/MSC, 2005 ▶); cell refinement: *CrystalClear*; data reduction: *CrystalClear*; program(s) used to solve structure: *SHELXS97* (Sheldrick, 2008 ▶); program(s) used to refine structure: *SHELXL97* (Sheldrick, 2008 ▶); molecular graphics: *SHELXTL* (Sheldrick, 2008 ▶); software used to prepare material for publication: *SHELXTL*.

## Supplementary Material

Crystal structure: contains datablocks I, global. DOI: 10.1107/S1600536808022174/sj2514sup1.cif

Structure factors: contains datablocks I. DOI: 10.1107/S1600536808022174/sj2514Isup2.hkl

Additional supplementary materials:  crystallographic information; 3D view; checkCIF report

## Figures and Tables

**Fig. 1.**
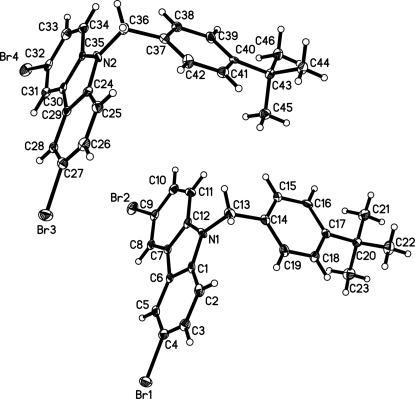
A view of the molecular structure of (I). Displacement ellipsoids are drawn at the 30% probability level (arbitrary spheres for H atoms).

## supplementary crystallographic information

### Comment

Carbazole derivatives substituted by *N*-alkylation possess valuable pharmaceutical properties (Buu-Hoï & Royer, 1950; Harfenist & Joyner, 1983; Caulfield *et al.*, 2002; Harper *et al.*, 2002). In this paper, the structure of 3,6-dibromo-9-(4-*tert*-butylbenzyl)-9*H*-carbazole, (I), which was synthesized by the *N*-alkylation of 1-*tert*-butyl-4-(chloromethyl)benzene with 3,6-dibromo-9*H*-carbazole  is reported, Fig. 1. The compound crystallises with two unique molecules in the asymmetric unit. Each carbazole ring system is essentially planar with mean deviations of 0.0077Å and 0.0089Å for the two molecules. In each molecule, the carbazole planes and make dihedral angles of 78.9 (2)° and 81.8 (2)° with the planes of the respective benzene rings. The C—Br distances fall in the range 1.894 (6) to 1.909 (5) Å, consistent with the literature (Allen *et al.*, 1987).

### Experimental

The title compound was prepared according to the procedures of Smith *et al.* (1992) and Duan *et al.* (2005).  The compound, (I) (40 mg), was dissolved in a mixture of chloroform (10 ml) and ethanol (5 ml) and the solution was kept at room temperature for 18 d. Natural evaporation of the solution gave colourless crystals suitable for X-Ray analysis (m.p. 434–435 K).

### Refinement

All H atoms were positioned geometrically and refined using a riding model, with C—H = 0.93Å and U_iso_(H) = 1.2U_eq_(C) for aromatic H atoms, C—H = 0.97Å and U_iso_(H) = 1.2U_eq_(C) for CH_2_ H atoms and C—H = 0.96Å and U_iso_(H) = 1.5U_eq_(C) for CH_3_ H atoms.

### Crystal data

**Table:**

C_23_H_21_Br_2_N	*Z* = 4
*M**_r_* = 471.23	*F*_000_ = 944
Triclinic, *P*1	*D*_x_ = 1.598 Mg m^−^^3^
Hall symbol: -P 1	Melting point = 434–435 K
*a* = 11.240 (2) Å	Mo *K*α radiation λ = 0.71073 Å
*b* = 12.921 (3) Å	Cell parameters from 3716 reflections
*c* = 15.694 (3) Å	θ = 1.5–27.9º
α = 105.43 (3)º	µ = 4.15 mm^−^^1^
β = 108.53 (3)º	*T* = 113 (2) K
γ = 103.09 (3)º	Prism, colorless
*V* = 1958.9 (10) Å^3^	0.10 × 0.08 × 0.04 mm

### Data collection

**Table:**

Rigaku Saturn CCD diffractometer	6891 independent reflections
Radiation source: rotating anode	4712 reflections with *I* > 2σ(*I*)
Monochromator: confocal	*R*_int_ = 0.051
*T* = 293(2) K	θ_max_ = 25.0º
ω scans	θ_min_ = 1.5º
Absorption correction: multi-scan(CrystalClear; Rigaku/MSC, 2005)	*h* = −9→13
*T*_min_ = 0.682, *T*_max_ = 0.852	*k* = −15→15
12085 measured reflections	*l* = −18→17

### Refinement

**Table:**

Refinement on *F*^2^	Secondary atom site location: difference Fourier map
Least-squares matrix: full	Hydrogen site location: inferred from neighbouring sites
*R*[*F*^2^ > 2σ(*F*^2^)] = 0.055	H-atom parameters constrained
*wR*(*F*^2^) = 0.112	*w* = 1/[σ^2^(*F*_o_^2^) + (0.0441*P*)^2^] where *P* = (*F*_o_^2^ + 2*F*_c_^2^)/3
*S* = 1.01	(Δ/σ)_max_ = 0.001
6891 reflections	Δρ_max_ = 0.46 e Å^−^^3^
475 parameters	Δρ_min_ = −0.46 e Å^−^^3^
Primary atom site location: structure-invariant direct methods	Extinction correction: none

### Special details

**Table:**

Geometry. All e.s.d.'s (except the e.s.d. in the dihedral angle between two l.s. planes) are estimated using the full covariance matrix. The cell e.s.d.'s are taken into account individually in the estimation of e.s.d.'s in distances, angles and torsion angles; correlations between e.s.d.'s in cell parameters are only used when they are defined by crystal symmetry. An approximate (isotropic) treatment of cell e.s.d.'s is used for estimating e.s.d.'s involving l.s. planes.
Refinement. Refinement of *F*^2^ against ALL reflections. The weighted *R*-factor *wR* and goodness of fit *S* are based on *F*^2^, conventional *R*-factors *R* are based on *F*, with *F* set to zero for negative *F*^2^. The threshold expression of *F*^2^ > σ(*F*^2^) is used only for calculating *R*-factors(gt) *etc*. and is not relevant to the choice of reflections for refinement. *R*-factors based on *F*^2^ are statistically about twice as large as those based on *F*, and *R*- factors based on ALL data will be even larger.

### Fractional atomic coordinates and isotropic or equivalent isotropic displacement parameters (Å^2^)

**Table:**

	*x*	*y*	*z*	*U*_iso_*/*U*_eq_	
Br1	0.96240 (7)	0.27334 (4)	1.22201 (4)	0.03941 (19)	
Br2	1.17197 (7)	0.38918 (5)	0.76081 (4)	0.04177 (19)	
Br3	0.40645 (7)	−0.22495 (4)	0.71466 (4)	0.04018 (19)	
Br4	0.72986 (7)	−0.12045 (5)	0.29922 (4)	0.03927 (18)	
N1	0.7041 (5)	0.3752 (3)	0.8695 (3)	0.0221 (10)	
N2	0.2243 (5)	−0.1201 (3)	0.3530 (3)	0.0215 (10)	
C1	0.7459 (6)	0.3489 (4)	0.9523 (3)	0.0250 (13)	
C2	0.6817 (6)	0.3308 (4)	1.0116 (4)	0.0297 (14)	
H2	0.5964	0.3345	0.9986	0.036*	
C3	0.7475 (6)	0.3070 (4)	1.0907 (4)	0.0288 (14)	
H3	0.7063	0.2949	1.1318	0.035*	
C4	0.8725 (7)	0.3011 (4)	1.1094 (3)	0.0304 (15)	
C5	0.9398 (6)	0.3174 (3)	1.0509 (3)	0.0237 (12)	
H5	1.0248	0.3127	1.0645	0.028*	
C6	0.8735 (6)	0.3410 (3)	0.9708 (3)	0.0216 (13)	
C7	0.9125 (6)	0.3640 (4)	0.8957 (3)	0.0217 (12)	
C8	1.0244 (6)	0.3672 (3)	0.8753 (3)	0.0240 (13)	
H8	1.0964	0.3540	0.9146	0.029*	
C9	1.0251 (6)	0.3906 (4)	0.7951 (4)	0.0277 (14)	
C10	0.9171 (6)	0.4106 (4)	0.7345 (4)	0.0292 (15)	
H10	0.9208	0.4257	0.6805	0.035*	
C11	0.8074 (6)	0.4081 (4)	0.7545 (3)	0.0254 (13)	
H11	0.7360	0.4216	0.7150	0.031*	
C12	0.8049 (6)	0.3847 (3)	0.8359 (3)	0.0229 (13)	
C13	0.5880 (6)	0.4076 (4)	0.8353 (4)	0.0336 (15)	
H13A	0.5196	0.3697	0.8527	0.040*	
H13B	0.5529	0.3822	0.7654	0.040*	
C14	0.6203 (6)	0.5352 (4)	0.8772 (3)	0.0232 (12)	
C15	0.6477 (6)	0.6037 (4)	0.8271 (3)	0.0320 (15)	
H15	0.6390	0.5698	0.7642	0.038*	
C16	0.6884 (6)	0.7231 (4)	0.8683 (3)	0.0289 (14)	
H16	0.7070	0.7672	0.8329	0.035*	
C17	0.7008 (5)	0.7753 (4)	0.9615 (3)	0.0198 (12)	
C18	0.6714 (6)	0.7060 (4)	1.0113 (4)	0.0250 (13)	
H18	0.6781	0.7395	1.0737	0.030*	
C19	0.6323 (6)	0.5881 (4)	0.9705 (4)	0.0283 (14)	
H19	0.6141	0.5440	1.0060	0.034*	
C20	0.7512 (6)	0.9073 (4)	1.0123 (3)	0.0233 (13)	
C21	0.7776 (7)	0.9676 (4)	0.9457 (4)	0.0401 (17)	
H21A	0.8144	1.0487	0.9806	0.060*	
H21B	0.6954	0.9496	0.8920	0.060*	
H21C	0.8396	0.9428	0.9225	0.060*	
C22	0.6453 (7)	0.9413 (4)	1.0432 (4)	0.0412 (17)	
H22A	0.6290	0.9043	1.0860	0.062*	
H22B	0.5642	0.9184	0.9872	0.062*	
H22C	0.6763	1.0226	1.0759	0.062*	
C23	0.8795 (6)	0.9417 (4)	1.1000 (4)	0.0365 (16)	
H23A	0.9436	0.9160	1.0801	0.055*	
H23B	0.8620	0.9076	1.1441	0.055*	
H23C	0.9144	1.0233	1.1313	0.055*	
C24	0.2497 (6)	−0.1459 (3)	0.4368 (3)	0.0232 (13)	
C25	0.1684 (6)	−0.1601 (4)	0.4870 (3)	0.0267 (13)	
H25	0.0844	−0.1531	0.4661	0.032*	
C26	0.2170 (7)	−0.1848 (4)	0.5688 (4)	0.0312 (15)	
H26	0.1651	−0.1951	0.6037	0.037*	
C27	0.3413 (6)	−0.1944 (4)	0.5992 (3)	0.0242 (13)	
C28	0.4236 (6)	−0.1816 (3)	0.5510 (3)	0.0225 (13)	
H28	0.5070	−0.1895	0.5727	0.027*	
C29	0.3755 (6)	−0.1560 (4)	0.4675 (3)	0.0202 (12)	
C30	0.4306 (6)	−0.1369 (3)	0.3996 (3)	0.0209 (12)	
C31	0.5495 (6)	−0.1379 (4)	0.3913 (3)	0.0244 (13)	
H31	0.6138	−0.1528	0.4361	0.029*	
C32	0.5699 (6)	−0.1164 (4)	0.3150 (4)	0.0253 (13)	
C33	0.4756 (6)	−0.0936 (4)	0.2466 (4)	0.0267 (13)	
H33	0.4931	−0.0787	0.1961	0.032*	
C34	0.3577 (6)	−0.0931 (3)	0.2537 (3)	0.0257 (14)	
H34	0.2938	−0.0791	0.2080	0.031*	
C35	0.3351 (6)	−0.1141 (3)	0.3311 (3)	0.0185 (12)	
C36	0.1124 (6)	−0.0882 (3)	0.3058 (3)	0.0249 (13)	
H36A	0.0335	−0.1303	0.3111	0.030*	
H36B	0.0947	−0.1087	0.2378	0.030*	
C37	0.1396 (6)	0.0393 (4)	0.3505 (3)	0.0245 (13)	
C38	0.2053 (6)	0.1155 (4)	0.3192 (3)	0.0256 (13)	
H38	0.2276	0.0881	0.2674	0.031*	
C39	0.2386 (6)	0.2327 (4)	0.3642 (3)	0.0268 (13)	
H39	0.2827	0.2821	0.3418	0.032*	
C40	0.2080 (5)	0.2775 (4)	0.4414 (3)	0.0188 (11)	
C41	0.1410 (6)	0.1999 (4)	0.4720 (3)	0.0266 (13)	
H41	0.1189	0.2270	0.5240	0.032*	
C42	0.1066 (6)	0.0831 (4)	0.4264 (4)	0.0281 (13)	
H42	0.0602	0.0334	0.4475	0.034*	
C43	0.2505 (6)	0.4068 (4)	0.4975 (3)	0.0218 (12)	
C44	0.1313 (6)	0.4382 (4)	0.5087 (4)	0.0309 (14)	
H44A	0.0954	0.3954	0.5417	0.046*	
H44B	0.0639	0.4204	0.4460	0.046*	
H44C	0.1600	0.5183	0.5453	0.046*	
C45	0.3586 (6)	0.4352 (4)	0.5971 (3)	0.0304 (14)	
H45A	0.3235	0.3919	0.6300	0.046*	
H45B	0.3866	0.5153	0.6339	0.046*	
H45C	0.4335	0.4164	0.5897	0.046*	
C46	0.3061 (6)	0.4770 (4)	0.4454 (4)	0.0323 (15)	
H46A	0.3331	0.5569	0.4823	0.048*	
H46B	0.2385	0.4589	0.3827	0.048*	
H46C	0.3817	0.4592	0.4384	0.048*	

### Atomic displacement parameters (Å^2^)

**Table:**

	*U*^11^	*U*^22^	*U*^33^	*U*^12^	*U*^13^	*U*^23^
Br1	0.0530 (5)	0.0296 (3)	0.0276 (3)	0.0098 (3)	0.0069 (3)	0.0133 (2)
Br2	0.0489 (5)	0.0399 (3)	0.0429 (3)	0.0163 (3)	0.0301 (4)	0.0095 (3)
Br3	0.0521 (5)	0.0372 (3)	0.0297 (3)	0.0113 (3)	0.0126 (3)	0.0177 (2)
Br4	0.0338 (5)	0.0463 (3)	0.0379 (3)	0.0149 (3)	0.0215 (3)	0.0062 (3)
N1	0.014 (3)	0.0200 (19)	0.025 (2)	0.007 (2)	−0.001 (2)	0.0091 (17)
N2	0.023 (3)	0.0172 (19)	0.028 (2)	0.011 (2)	0.007 (2)	0.0121 (17)
C1	0.033 (4)	0.012 (2)	0.030 (3)	0.010 (3)	0.012 (3)	0.005 (2)
C2	0.031 (4)	0.018 (2)	0.037 (3)	0.007 (3)	0.013 (3)	0.009 (2)
C3	0.030 (4)	0.024 (2)	0.032 (3)	0.007 (3)	0.015 (3)	0.008 (2)
C4	0.044 (5)	0.016 (2)	0.024 (3)	0.005 (3)	0.010 (3)	0.005 (2)
C5	0.021 (4)	0.015 (2)	0.028 (3)	0.007 (2)	0.004 (3)	0.004 (2)
C6	0.025 (4)	0.010 (2)	0.021 (2)	0.005 (2)	0.004 (3)	0.0003 (19)
C7	0.024 (4)	0.013 (2)	0.021 (2)	0.006 (2)	0.003 (3)	0.0018 (19)
C8	0.026 (4)	0.015 (2)	0.023 (3)	0.006 (2)	0.005 (3)	0.001 (2)
C9	0.036 (4)	0.015 (2)	0.030 (3)	0.006 (3)	0.018 (3)	0.003 (2)
C10	0.045 (5)	0.016 (2)	0.026 (3)	0.005 (3)	0.018 (3)	0.007 (2)
C11	0.023 (4)	0.019 (2)	0.022 (3)	0.001 (2)	−0.001 (3)	0.007 (2)
C12	0.029 (4)	0.012 (2)	0.026 (3)	0.005 (2)	0.013 (3)	0.002 (2)
C13	0.030 (4)	0.025 (3)	0.034 (3)	0.007 (3)	0.002 (3)	0.009 (2)
C14	0.012 (4)	0.022 (2)	0.026 (3)	0.005 (2)	−0.001 (3)	0.005 (2)
C15	0.046 (5)	0.026 (3)	0.023 (3)	0.022 (3)	0.009 (3)	0.008 (2)
C16	0.035 (4)	0.028 (3)	0.022 (3)	0.012 (3)	0.003 (3)	0.015 (2)
C17	0.012 (3)	0.020 (2)	0.023 (2)	0.005 (2)	0.003 (3)	0.007 (2)
C18	0.025 (4)	0.027 (3)	0.027 (3)	0.011 (3)	0.015 (3)	0.009 (2)
C19	0.032 (4)	0.024 (2)	0.038 (3)	0.013 (3)	0.020 (3)	0.016 (2)
C20	0.023 (4)	0.018 (2)	0.021 (2)	0.004 (2)	0.003 (3)	0.003 (2)
C21	0.063 (6)	0.019 (2)	0.034 (3)	0.009 (3)	0.019 (4)	0.009 (2)
C22	0.048 (5)	0.031 (3)	0.049 (4)	0.017 (3)	0.024 (4)	0.012 (3)
C23	0.036 (5)	0.024 (3)	0.034 (3)	−0.001 (3)	0.007 (3)	0.004 (2)
C24	0.026 (4)	0.012 (2)	0.019 (2)	0.000 (2)	0.001 (3)	0.0006 (19)
C25	0.022 (4)	0.022 (2)	0.028 (3)	0.005 (2)	0.006 (3)	0.005 (2)
C26	0.039 (5)	0.025 (3)	0.032 (3)	0.008 (3)	0.020 (3)	0.010 (2)
C27	0.023 (4)	0.017 (2)	0.028 (3)	0.002 (2)	0.007 (3)	0.008 (2)
C28	0.022 (4)	0.015 (2)	0.023 (2)	0.005 (2)	0.004 (3)	0.003 (2)
C29	0.019 (4)	0.015 (2)	0.019 (2)	0.004 (2)	0.004 (3)	0.0010 (19)
C30	0.026 (4)	0.012 (2)	0.024 (3)	0.006 (2)	0.012 (3)	0.0015 (19)
C31	0.028 (4)	0.019 (2)	0.021 (2)	0.007 (2)	0.006 (3)	0.004 (2)
C32	0.025 (4)	0.014 (2)	0.030 (3)	0.003 (2)	0.013 (3)	−0.002 (2)
C33	0.028 (4)	0.020 (2)	0.030 (3)	0.002 (3)	0.014 (3)	0.006 (2)
C34	0.034 (4)	0.016 (2)	0.022 (3)	0.004 (3)	0.010 (3)	0.007 (2)
C35	0.020 (4)	0.011 (2)	0.020 (2)	0.007 (2)	0.006 (3)	0.0008 (19)
C36	0.027 (4)	0.016 (2)	0.026 (3)	0.007 (2)	0.009 (3)	0.002 (2)
C37	0.024 (4)	0.026 (2)	0.023 (3)	0.012 (3)	0.006 (3)	0.009 (2)
C38	0.032 (4)	0.031 (3)	0.021 (2)	0.017 (3)	0.014 (3)	0.012 (2)
C39	0.036 (4)	0.024 (2)	0.030 (3)	0.014 (3)	0.016 (3)	0.017 (2)
C40	0.009 (3)	0.023 (2)	0.014 (2)	0.006 (2)	−0.005 (2)	0.0032 (19)
C41	0.027 (4)	0.029 (3)	0.026 (3)	0.010 (3)	0.012 (3)	0.011 (2)
C42	0.024 (4)	0.022 (2)	0.041 (3)	0.005 (3)	0.016 (3)	0.013 (2)
C43	0.019 (4)	0.021 (2)	0.023 (2)	0.005 (2)	0.009 (3)	0.005 (2)
C44	0.036 (5)	0.026 (3)	0.031 (3)	0.013 (3)	0.015 (3)	0.007 (2)
C45	0.032 (4)	0.027 (3)	0.027 (3)	0.008 (3)	0.008 (3)	0.009 (2)
C46	0.042 (5)	0.022 (2)	0.033 (3)	0.013 (3)	0.015 (3)	0.010 (2)

### Geometric parameters (Å, °)

**Table:**

Br1—C4	1.908 (5)	C22—H22B	0.9600
Br2—C9	1.894 (6)	C22—H22C	0.9600
Br3—C27	1.909 (5)	C23—H23A	0.9600
Br4—C32	1.901 (6)	C23—H23B	0.9600
N1—C12	1.388 (7)	C23—H23C	0.9600
N1—C1	1.395 (6)	C24—C29	1.391 (8)
N1—C13	1.445 (7)	C24—C25	1.397 (7)
N2—C35	1.384 (6)	C25—C26	1.379 (7)
N2—C24	1.399 (6)	C25—H25	0.9300
N2—C36	1.452 (7)	C26—C27	1.372 (8)
C1—C2	1.381 (7)	C26—H26	0.9300
C1—C6	1.403 (8)	C27—C28	1.377 (7)
C2—C3	1.380 (7)	C28—C29	1.408 (6)
C2—H2	0.9300	C28—H28	0.9300
C3—C4	1.366 (8)	C29—C30	1.438 (6)
C3—H3	0.9300	C30—C31	1.386 (7)
C4—C5	1.390 (7)	C30—C35	1.406 (7)
C5—C6	1.393 (7)	C31—C32	1.374 (6)
C5—H5	0.9300	C31—H31	0.9300
C6—C7	1.457 (6)	C32—C33	1.396 (8)
C7—C8	1.388 (7)	C33—C34	1.367 (8)
C7—C12	1.405 (8)	C33—H33	0.9300
C8—C9	1.372 (6)	C34—C35	1.398 (6)
C8—H8	0.9300	C34—H34	0.9300
C9—C10	1.409 (8)	C36—C37	1.522 (6)
C10—C11	1.362 (8)	C36—H36A	0.9700
C10—H10	0.9300	C36—H36B	0.9700
C11—C12	1.396 (6)	C37—C42	1.377 (6)
C11—H11	0.9300	C37—C38	1.384 (6)
C13—C14	1.512 (6)	C38—C39	1.392 (6)
C13—H13A	0.9700	C38—H38	0.9300
C13—H13B	0.9700	C39—C40	1.379 (6)
C14—C15	1.376 (6)	C39—H39	0.9300
C14—C19	1.389 (6)	C40—C41	1.396 (6)
C15—C16	1.403 (6)	C40—C43	1.544 (6)
C15—H15	0.9300	C41—C42	1.387 (6)
C16—C17	1.385 (6)	C41—H41	0.9300
C16—H16	0.9300	C42—H42	0.9300
C17—C18	1.386 (6)	C43—C45	1.529 (7)
C17—C20	1.554 (6)	C43—C44	1.531 (7)
C18—C19	1.387 (6)	C43—C46	1.531 (6)
C18—H18	0.9300	C44—H44A	0.9600
C19—H19	0.9300	C44—H44B	0.9600
C20—C23	1.515 (8)	C44—H44C	0.9600
C20—C21	1.521 (6)	C45—H45A	0.9600
C20—C22	1.529 (8)	C45—H45B	0.9600
C21—H21A	0.9600	C45—H45C	0.9600
C21—H21B	0.9600	C46—H46A	0.9600
C21—H21C	0.9600	C46—H46B	0.9600
C22—H22A	0.9600	C46—H46C	0.9600
			
C12—N1—C1	108.1 (5)	C20—C23—H23C	109.5
C12—N1—C13	125.4 (4)	H23A—C23—H23C	109.5
C1—N1—C13	125.6 (5)	H23B—C23—H23C	109.5
C35—N2—C24	107.4 (5)	C29—C24—C25	121.7 (5)
C35—N2—C36	125.4 (4)	C29—C24—N2	109.5 (4)
C24—N2—C36	126.5 (4)	C25—C24—N2	128.8 (6)
C2—C1—N1	130.2 (6)	C26—C25—C24	117.6 (6)
C2—C1—C6	121.0 (5)	C26—C25—H25	121.2
N1—C1—C6	108.8 (4)	C24—C25—H25	121.2
C3—C2—C1	118.2 (6)	C27—C26—C25	120.6 (5)
C3—C2—H2	120.9	C27—C26—H26	119.7
C1—C2—H2	120.9	C25—C26—H26	119.7
C4—C3—C2	120.7 (5)	C26—C27—C28	123.2 (5)
C4—C3—H3	119.6	C26—C27—Br3	119.2 (4)
C2—C3—H3	119.6	C28—C27—Br3	117.6 (4)
C3—C4—C5	122.8 (5)	C27—C28—C29	116.9 (5)
C3—C4—Br1	119.4 (4)	C27—C28—H28	121.5
C5—C4—Br1	117.8 (5)	C29—C28—H28	121.5
C4—C5—C6	116.6 (6)	C24—C29—C28	119.9 (5)
C4—C5—H5	121.7	C24—C29—C30	107.0 (4)
C6—C5—H5	121.7	C28—C29—C30	133.0 (5)
C5—C6—C1	120.7 (5)	C31—C30—C35	119.9 (4)
C5—C6—C7	131.9 (5)	C31—C30—C29	133.6 (5)
C1—C6—C7	107.4 (4)	C35—C30—C29	106.5 (5)
C8—C7—C12	120.5 (4)	C32—C31—C30	118.2 (5)
C8—C7—C6	134.0 (5)	C32—C31—H31	120.9
C12—C7—C6	105.5 (5)	C30—C31—H31	120.9
C9—C8—C7	117.6 (5)	C31—C32—C33	122.3 (5)
C9—C8—H8	121.2	C31—C32—Br4	119.5 (4)
C7—C8—H8	121.2	C33—C32—Br4	118.2 (4)
C8—C9—C10	122.1 (5)	C34—C33—C32	120.0 (5)
C8—C9—Br2	119.3 (5)	C34—C33—H33	120.0
C10—C9—Br2	118.5 (4)	C32—C33—H33	120.0
C11—C10—C9	120.4 (5)	C33—C34—C35	118.7 (5)
C11—C10—H10	119.8	C33—C34—H34	120.7
C9—C10—H10	119.8	C35—C34—H34	120.7
C10—C11—C12	118.3 (5)	N2—C35—C34	129.6 (5)
C10—C11—H11	120.8	N2—C35—C30	109.5 (4)
C12—C11—H11	120.8	C34—C35—C30	120.9 (5)
N1—C12—C11	128.8 (5)	N2—C36—C37	111.7 (4)
N1—C12—C7	110.2 (4)	N2—C36—H36A	109.3
C11—C12—C7	121.0 (5)	C37—C36—H36A	109.3
N1—C13—C14	111.9 (5)	N2—C36—H36B	109.3
N1—C13—H13A	109.2	C37—C36—H36B	109.3
C14—C13—H13A	109.2	H36A—C36—H36B	107.9
N1—C13—H13B	109.2	C42—C37—C38	117.8 (4)
C14—C13—H13B	109.2	C42—C37—C36	121.4 (4)
H13A—C13—H13B	107.9	C38—C37—C36	120.7 (4)
C15—C14—C19	117.8 (4)	C37—C38—C39	120.9 (4)
C15—C14—C13	121.3 (4)	C37—C38—H38	119.6
C19—C14—C13	120.7 (4)	C39—C38—H38	119.6
C14—C15—C16	121.9 (4)	C40—C39—C38	121.7 (4)
C14—C15—H15	119.1	C40—C39—H39	119.1
C16—C15—H15	119.1	C38—C39—H39	119.1
C17—C16—C15	120.1 (4)	C39—C40—C41	116.9 (4)
C17—C16—H16	120.0	C39—C40—C43	123.5 (4)
C15—C16—H16	120.0	C41—C40—C43	119.4 (4)
C16—C17—C18	117.9 (4)	C42—C41—C40	121.3 (4)
C16—C17—C20	122.3 (4)	C42—C41—H41	119.4
C18—C17—C20	119.8 (4)	C40—C41—H41	119.4
C17—C18—C19	121.8 (4)	C37—C42—C41	121.3 (4)
C17—C18—H18	119.1	C37—C42—H42	119.3
C19—C18—H18	119.1	C41—C42—H42	119.3
C18—C19—C14	120.6 (4)	C45—C43—C44	109.8 (4)
C18—C19—H19	119.7	C45—C43—C46	109.4 (4)
C14—C19—H19	119.7	C44—C43—C46	108.2 (4)
C23—C20—C21	109.3 (5)	C45—C43—C40	107.6 (4)
C23—C20—C22	110.1 (4)	C44—C43—C40	110.5 (4)
C21—C20—C22	109.0 (4)	C46—C43—C40	111.3 (4)
C23—C20—C17	108.0 (4)	C43—C44—H44A	109.5
C21—C20—C17	111.7 (4)	C43—C44—H44B	109.5
C22—C20—C17	108.8 (4)	H44A—C44—H44B	109.5
C20—C21—H21A	109.5	C43—C44—H44C	109.5
C20—C21—H21B	109.5	H44A—C44—H44C	109.5
H21A—C21—H21B	109.5	H44B—C44—H44C	109.5
C20—C21—H21C	109.5	C43—C45—H45A	109.5
H21A—C21—H21C	109.5	C43—C45—H45B	109.5
H21B—C21—H21C	109.5	H45A—C45—H45B	109.5
C20—C22—H22A	109.5	C43—C45—H45C	109.5
C20—C22—H22B	109.5	H45A—C45—H45C	109.5
H22A—C22—H22B	109.5	H45B—C45—H45C	109.5
C20—C22—H22C	109.5	C43—C46—H46A	109.5
H22A—C22—H22C	109.5	C43—C46—H46B	109.5
H22B—C22—H22C	109.5	H46A—C46—H46B	109.5
C20—C23—H23A	109.5	C43—C46—H46C	109.5
C20—C23—H23B	109.5	H46A—C46—H46C	109.5
H23A—C23—H23B	109.5	H46B—C46—H46C	109.5
			
C12—N1—C1—C2	−179.9 (4)	C35—N2—C24—C29	0.0 (5)
C13—N1—C1—C2	10.5 (8)	C36—N2—C24—C29	−171.3 (4)
C12—N1—C1—C6	−0.3 (5)	C35—N2—C24—C25	179.6 (4)
C13—N1—C1—C6	−169.9 (4)	C36—N2—C24—C25	8.3 (7)
N1—C1—C2—C3	−179.2 (5)	C29—C24—C25—C26	−0.1 (7)
C6—C1—C2—C3	1.3 (7)	N2—C24—C25—C26	−179.6 (4)
C1—C2—C3—C4	−0.3 (7)	C24—C25—C26—C27	0.3 (7)
C2—C3—C4—C5	−0.5 (8)	C25—C26—C27—C28	−0.7 (8)
C2—C3—C4—Br1	177.8 (3)	C25—C26—C27—Br3	178.4 (3)
C3—C4—C5—C6	0.3 (7)	C26—C27—C28—C29	0.8 (7)
Br1—C4—C5—C6	−178.1 (3)	Br3—C27—C28—C29	−178.3 (3)
C4—C5—C6—C1	0.7 (6)	C25—C24—C29—C28	0.2 (7)
C4—C5—C6—C7	179.1 (4)	N2—C24—C29—C28	179.9 (4)
C2—C1—C6—C5	−1.5 (7)	C25—C24—C29—C30	179.8 (4)
N1—C1—C6—C5	178.9 (4)	N2—C24—C29—C30	−0.6 (5)
C2—C1—C6—C7	179.7 (4)	C27—C28—C29—C24	−0.6 (6)
N1—C1—C6—C7	0.1 (5)	C27—C28—C29—C30	−180.0 (4)
C5—C6—C7—C8	2.2 (8)	C24—C29—C30—C31	−178.7 (5)
C1—C6—C7—C8	−179.2 (5)	C28—C29—C30—C31	0.8 (9)
C5—C6—C7—C12	−178.5 (5)	C24—C29—C30—C35	0.9 (5)
C1—C6—C7—C12	0.1 (5)	C28—C29—C30—C35	−179.6 (5)
C12—C7—C8—C9	−0.3 (6)	C35—C30—C31—C32	−0.1 (6)
C6—C7—C8—C9	178.9 (4)	C29—C30—C31—C32	179.5 (4)
C7—C8—C9—C10	−0.1 (7)	C30—C31—C32—C33	0.1 (7)
C7—C8—C9—Br2	−177.2 (3)	C30—C31—C32—Br4	−178.5 (3)
C8—C9—C10—C11	0.5 (7)	C31—C32—C33—C34	−0.5 (7)
Br2—C9—C10—C11	177.5 (3)	Br4—C32—C33—C34	178.1 (3)
C9—C10—C11—C12	−0.3 (7)	C32—C33—C34—C35	0.9 (7)
C1—N1—C12—C11	178.9 (4)	C24—N2—C35—C34	178.6 (4)
C13—N1—C12—C11	−11.5 (7)	C36—N2—C35—C34	−9.9 (7)
C1—N1—C12—C7	0.4 (5)	C24—N2—C35—C30	0.6 (5)
C13—N1—C12—C7	170.0 (4)	C36—N2—C35—C30	172.0 (4)
C10—C11—C12—N1	−178.5 (4)	C33—C34—C35—N2	−178.7 (4)
C10—C11—C12—C7	−0.1 (7)	C33—C34—C35—C30	−0.9 (6)
C8—C7—C12—N1	179.1 (4)	C31—C30—C35—N2	178.7 (4)
C6—C7—C12—N1	−0.3 (5)	C29—C30—C35—N2	−0.9 (5)
C8—C7—C12—C11	0.5 (7)	C31—C30—C35—C34	0.5 (7)
C6—C7—C12—C11	−179.0 (4)	C29—C30—C35—C34	−179.2 (4)
C12—N1—C13—C14	−78.4 (6)	C35—N2—C36—C37	−84.9 (5)
C1—N1—C13—C14	89.4 (6)	C24—N2—C36—C37	85.0 (6)
N1—C13—C14—C15	96.5 (6)	N2—C36—C37—C42	−90.0 (6)
N1—C13—C14—C19	−78.9 (7)	N2—C36—C37—C38	86.4 (6)
C19—C14—C15—C16	0.8 (9)	C42—C37—C38—C39	1.0 (8)
C13—C14—C15—C16	−174.7 (6)	C36—C37—C38—C39	−175.5 (5)
C14—C15—C16—C17	−0.6 (9)	C37—C38—C39—C40	0.0 (9)
C15—C16—C17—C18	−0.2 (9)	C38—C39—C40—C41	−0.4 (8)
C15—C16—C17—C20	177.3 (5)	C38—C39—C40—C43	176.1 (5)
C16—C17—C18—C19	0.8 (9)	C39—C40—C41—C42	−0.3 (8)
C20—C17—C18—C19	−176.8 (5)	C43—C40—C41—C42	−176.9 (5)
C17—C18—C19—C14	−0.5 (9)	C38—C37—C42—C41	−1.7 (9)
C15—C14—C19—C18	−0.3 (9)	C36—C37—C42—C41	174.8 (5)
C13—C14—C19—C18	175.3 (5)	C40—C41—C42—C37	1.4 (9)
C16—C17—C20—C23	−117.6 (6)	C39—C40—C43—C45	−109.0 (6)
C18—C17—C20—C23	59.8 (6)	C41—C40—C43—C45	67.4 (6)
C16—C17—C20—C21	2.6 (8)	C39—C40—C43—C44	131.1 (5)
C18—C17—C20—C21	−180.0 (5)	C41—C40—C43—C44	−52.5 (6)
C16—C17—C20—C22	122.9 (6)	C39—C40—C43—C46	10.8 (7)
C18—C17—C20—C22	−59.7 (6)	C41—C40—C43—C46	−172.8 (5)

## References

[bb1] Allen, F. H., Kennard, O., Watson, D. G., Brammer, L., Orpen, A. G. & Taylor, R. (1987). *J. Chem. Soc. Perkin Trans. 2*, pp. S1–19.

[bb3] Buu-Hoï, N. P. & Royer, R. (1950). *J. Org. Chem.* **15**, 123–130.

[bb4] Caulfield, T., Cherrier, M. P., Combeau, C. & Mailliet, P. (2002). European Patent 1253141.

[bb5] Duan, X. M., Han, J., Chen, L. G., Xu, Y. J. & Li, Y. (2005). *Fine Chem.* **22**, 39–40, 52.

[bb6] Harfenist, M. & Joyner, C. T. (1983). US Patent No. 4 379 160.

[bb7] Harper, R. W., Lin, H. S. & Richett, M. E. (2002). World Patent No. 02079154.

[bb8] Rigaku/MSC (2005). *CrystalClear* Rigaku/MSC, The Woodlands, Texas, USA.

[bb9] Sheldrick, G. M. (2008). *Acta Cryst.* A**64**, 112–122. 10.1107/S0108767307043930 18156677

[bb10] Smith, K., James, D. M., Mistry, A. G., Bye, M. R. & Faulkner, D. J. (1992). *Tetrahedron*, **48**, 7479–7488.

